# Fatal inanition in reindeer (*Rangifer tarandus tarandus*): Pathological findings in completely emaciated carcasses

**DOI:** 10.1186/1751-0147-49-27

**Published:** 2007-09-28

**Authors:** Terje D Josefsen, Karen K Sørensen, Torill Mørk, Svein D Mathiesen, Kathrine A Ryeng

**Affiliations:** 1 National Veterinary Institute Tromsø, Stakkevollvn. 23b, NO-9292 Tromsø, Norway; 2 Section of Arctic Veterinary Medicine, Department of Food Safety and Infection Biology, The Norwegian School of Veterinary Science, PO Box 6204, NO-9292 Tromsø, Norway; 3 Department of Cell Biology and Histology, Institute of Medical Biology, University of Tromsø, NO-9037 Tromsø, Norway

## Abstract

**Background:**

In a project to determine the causes of winter mortality in reindeer in Finnmark County, northern Norway, the most frequent diagnosis turned out to be complete emaciation, despite several of the reindeer having been given silage for up to 4 weeks before they died. The present paper describes autopsy results and other findings in these animals.

**Methods:**

Autopsies were made of 32 reindeer carcasses, and 28 of these were diagnosed as completely emaciated based on lack of visible fat and serous atrophy of subepicardial and bone marrow fat. Other investigations of the carcasses included histology, bacteriology, parasitology (counting of macro parasites and faecal egg counting), analysis of vitamin E and selenium in liver, chemical and botanical analysis of rumen content, analysis of lipid content in femur bone marrow and estimation of muscle atrophy by use of a muscle index.

**Results:**

Main findings were: Low carcass weight, severe muscle atrophy, hemosiderosis in liver and spleen, subcutaneous oedema (18%) and effusions to body cavities (18%). Two types of lipofuscin granula were identified in the liver: One type occurred in liver endothelial cells of all carcasses, while the other type occurred in hepatocytes, and prevailed in adult animals. Abomasal haemorrhages, consistent with previously described stress lesions, was present in 68% of the carcasses. Diarrhoea occurred in 2 cases, and loose faecal consistency was associated with silage feeding. Rumen content was low in crude protein. Grass dominated rumen content in silage-fed carcasses, while reindeer on natural pastures had mainly woody plants, mosses and litter in rumen. Stem dominated the grass fraction in rumens with high grass content, indicating ruminal indigestion as a cause of emaciation in silage fed animals. Some cases had heavy infestation of parasites such as warble fly larvae (*Hypoderma tarandi*), throat bot larvae (*Cephenemyiae trompe*) and lung nematodes.

**Conclusion:**

Lack of appropriate amounts and/or appropriate quality of feed has been the main cause of emaciation, though heavy infestation of parasites may have contributed to the emaciation in some cases.

## Background

Free-living reindeer are subjected to seasonal changes in food quality and availability, and weight loss in winter due to sub-maintenance feed intake, inanition, is regarded as normal. If inanition is severe and prolonged, the outcome will be fatal. When all fat depots are completely depleted the animals succumb in a cachectic state due to lack of energy to maintain body homeostasis.

Autopsies of such cases reveal severe muscle atrophy and a complete lack of visible fat – "complete emaciation" – as a main finding. Serous atrophy of subepicardial and bone marrow fat is considered a significant indication of lack of energy as the immediate cause of death [1].

In the experience of the National Veterinary Institute in Tromsø, Norway, death due to inanition is by far the most common diagnosis in reindeer that is found dead in winter without signs of trauma. These deaths occur most frequently in late winter, and are usually associated with heavy snow cover or icy crust that makes the pastures unavailable. Heavy parasitic burden may contribute to the disaster, but all together lack of food seems to be the main cause of emaciation in these cases.

Even though deaths due to inanition are considered common in reindeer, accurate descriptions of autopsy findings are lacking. Also, there are case reports of mortality in reindeer, in which complete emaciation has been a main finding at autopsy, but where the cause of emaciation is disputable. Such cases include mortality during winter-feeding with lichens and hay [2], and reindeer dying while kept for several years on a restricted area of cultivated grassland [3].

We here present the results from an expanded examination of 28 completely emaciated reindeer carcasses that originate from a pilot project aimed to determine causes of winter mortality in reindeer in Finnmark County, Norway. Reindeer owners in 3 herds were stimulated to collect carcasses that were found in the field in winter and spring, more or less intact (not eaten by predators or scavengers). The most frequent diagnosis in this material turned out to be complete emaciation, despite the fact that several of the reindeer had been given feed (mainly silage) for up to 4 weeks before they died.

The aim of the present paper is to describe pathological findings in reindeer succumbed to inanition in winter. We also investigated the gastrointestinal fill and rumen content in the same material of reindeer in order to contribute to the understanding of the seemingly paradox that reindeer die of emaciation during feeding.

## Methods

### Animals

Thirty-two frozen reindeer carcasses were received for autopsy from 3 herds (A, B and C) in Finnmark County, northern Norway. Twenty-eight of these were classified as completely emaciated at autopsy, based on three major macroscopic findings: no visible fat in the abdomen, serous atrophy of pericardial fat and orange to red, semi-transparent, gelatinous bone marrow in femur. The completely emaciated carcasses were found dead (n = 26) or euthanised in a moribund state (n = 2) in the period from the 8^th ^of March to the 24^th ^of May 2000 (late winter to early spring). The carcasses were frozen within 24 hours and kept frozen until autopsy, which were performed from April to September the same year.

The four not completely emaciated carcasses, 2 adult females and 2 yearlings, originated from Herds B and C, and are referred to as "control animals" throughout the text. Three of these had *in vivo *obtained bone fractures, and were either killed by wolverine or euthanised, whereas the cause of death was uncertain for one yearling.

The age distribution of completely emaciated carcasses was as follows: yearlings (9–12 mo): n = 17, two years old (21–24 mo): n = 4, adults (>33 months): n = 7. There were 11 male and 6 female yearlings, whereas all elder animals were females.

### Feeding history

The feeding history of the completely emaciated animals differed between herds:

Three of the 18 carcasses received from Herd A belonged to a group of calves that had been taken home for emergency feeding (baled grass silage and some lichens). These carcasses had been fed for 3–4 weeks before they died. The rest of the herd had been offered baled grass silage in the field (supplemental feeding), but there was no detailed information about whether individual animals actually had been eating of the silage, or even had access to the silage balls.

From Herd B was received 8 carcasses. Seven of these had been taken home and placed in a fenced area, where they had been fed mainly baled grass silage, supplemented by smaller amounts of lichens and reindeer pellets. Most of the animals had been fed about 2 weeks, while a single reindeer died after about 1 month of feeding.

From Herd C was received 2 carcasses. Both had been taken home for emergency feeding (grass silage, lichens, reindeer pellets). One succumbed within 1 day, the other died after one week of feeding.

### Autopsy

Autopsies were performed according to a standard operating procedure. Samples for histology were fixed in 10% buffered formalin, and histological sections stained routinely with haematoxilin and eosin (HE). All liver sections were stained with the Prussian blue technique for hemosiderin, and the Schmorl and Sudan Black B techniques for lipofuscins. Selected liver sections were stained with the Long Ziehl-Neelsen and Periodic Acid Schiff (PAS) techniques. Selected sections from kidney, spleen and lung were stained with Prussian blue.

Bacteriological cultivation of samples from liver, kidney and lung, and faecal examination for parasite eggs/larvae was performed in all autopsies. Bacteriological examination was done according to standard routines at the National Veterinary Institute, Norway. A modified McMaster technique using saturated NaCl solution with sucrose [4] was used to detect coccidia, helminth eggs and larvae in faeces. The detection level was 20 oocysts or eggs per gram (epg). The nematode eggs were identified to genus (*Capillaria *sp., *Nematodirus *sp., *Skrjabinema *sp.), or as "trichostrongyles". Larvae were regarded as those of the brainworm, *Elaphostrongylus rangiferi*, if they were of the typical size (0.3–0.4 mm) and had the characteristic dorsal spine over the tail. Similar-sized larvae without the dorsal spine over the tail, were classified as lungworm larvae, *Dictyocaulus *sp. Warble fly larvae (*Hypoderma tarandi*) and throat bot larvae (*Cephenemyia trompe*) were detected and counted from each individual during autopsy. Total carcass weight (TCW) was weighed to 0.5 kg on a mechanic spring scale (model 233, 0–300 kg, Salter, UK). Organs were weighed to 1 g on an electronic balance (HP-20K, 0.1 g–21 kg; A&D Instruments Ltd, UK). Some measurements are lacking in individual animals, due to accidents during transport and autopsy (missing hind leg in one carcass, accidental omission of registrations at autopsy, etc.).

### Vitamin E and selenium analyses

Samples (10 grams) of liver tissue from 27 of the 28 emaciated carcasses and the 4 control animals were frozen and later analysed for vitamin E and selenium content at the Section for Toxicology, National Veterinary Institute, Oslo. Vitamin E was analysed by high-performance liquid chromatography (HPLC), and selenium by atomic absorption spectroscopy, according to standard procedures at the National Veterinary Institute.

### Chemical analysis of rumen content

Representative samples of rumen content were frozen at -20°C, and samples from 19 carcasses, selected to be representative, were sent for analysis at the Norwegian Crop Research Institute, Holt Research Laboratory, Tromsø, Norway. Analysis of dry matter, ash and crude protein were performed according to Horwitz [5]. Dry matter content was determined after preheating for 24 hours at 80°C and heating for 103–105°C until constant weight. Ash content was determined by glowing at 550°C for 24 hours. Crude protein content was determined using Kjeldahl analysis, and multiplying Kjeldahl-N with 6.25. Plant fibre analysis was performed according to Van Soest [6-8]. Neutral detergent fiber (NDF), acid detergent fiber (ADF) and acid detergent lignin (ADL) were determined directly. Hemicelluloses were calculated as NDF minus ADF, celluloses as ADF minus ADL, and ADL was considered equivalent to lignin.

### Botanical analysis of rumen content

Representative samples of rumen content were fixed in an equal amount of 70% ethanol, and samples from 26 carcasses, selected to be representative, were sent to the Norwegian Institute for Nature Research (NINA), Trondheim, Norway, for analysis. The botanical composition was determined according to Gaare et al. [9]. Plant fragments were identified in 200 points regularly distributed on a 10-cm muslin frame with a stereomicroscope. Identification level was into 5 main groups: Grasses (graminoids), lichens, woody plants, mosses and litter. Grass fragments were categorised as either leaf or stem. Results were given as number of plant fragments in each group, in percent of the total number of plant fragments examined.

### Analysis of lipids in bone marrow

Samples from (right or left) femur bone marrow were collected from 27 of the 28 carcasses. The samples size varied from 1 to 15 g (mean 4.7 g). The samples were analysed at UniLab Analyse AS, Tromsø, Norway. Lipids were extracted from the samples according to Bligh & Dyer [10], and the lipid weight determined. Results are given as weight percentage of bone marrow wet weight.

### Calculation of muscle index

In 22 out of 28 completely emaciated carcasses a muscle index was calculated according to Tyler [11]. *Musculus gluteobiceps *was dissected out on one side, and weighed to 1 g. A subsample of the muscle was dried at 65°C to constant weight. The dry matter content of the subsample was determined, and the dry weight of the whole muscle was calculated. Greatest length of femur was measured to 1 mm. The muscle index was calculated using the formula: MI = M/(F)^3^, where MI = muscle index (kg/cm^3^), M = dry weight of *musculus gluteobiceps *(kg), and F = femur length (cm).

### Statistics

Results are expressed as mean values. Dispersion is expressed by use of standard deviation (SD) and range values. Groups were compared by use of the two sample t-test. The Pearsons linear correlation coefficient (r) was used for correlation estimates. All tests were performed as 2-tailed analyses and probabilities (p) less than 0.05 were considered statistically significant.

## Results

### General condition and reproductive status

According to the inclusion criteria the 28 completely emaciated carcasses had no visible fat in the abdomen, and showed serous atrophy of subepicardial fat and bone marrow. The distinction between complete emaciation (totally depleted fat depots) and severe emaciation appeared easy, and the macroscopic observations corresponded well with the results of analysis of fat content in femoral bone marrow (Table 1). Fat content in bone marrow (% of wet weight) was 1.0% or less in all 28 completely emaciated carcasses, while a severely emaciated male calf, still with visible fat in abdomen and pericardium, had 1.8% fat in bone marrow.

**Table 1:** Carcass weight, liver weight, fat content in femoral bone marrow, muscle index, and rumino-reticulum weights (tissue and content) of 28 completely emaciated reindeer carcasses. Mean (SD), range and number of animals (n). Total n less than 28 is due to lacking data.

	**Total carcass weight (TCW) (kg)**	**Liver weight (g)**	**Fat content in bone marrow (% of wet weight)**	**Muscle index (kg/cm3)**	**Rumino-reticulum weight (kg)**	**Rumino-reticulum weight (% of TCW)**
**Male yearlings**	25.7 (4.2)	385 (99)	0.6 (0.2)	3.8 (0.59)	4.2 (1.5)	17.1 (3.9)
	19–31.5	250–533	0.2–0.9	2.9–4.9	1.9–7.3	12.3–26.9
	n = 10	n = 11	n = 10	n = 8	n = 11	n = 10
**Female yearlings**	22.7 (2,8)	300 (40)	0.4 (0.1)	3.8 (0.70)	4.1 (1.2)	18.6 (4.0)
	18–26	264–354	0.3–0.5	3.0–4.5	2.8–5.3	14.6–23.0
	n = 6	n = 5	n = 6	n = 5	n = 5	n = 5
**Female two-year-old**	37.9 (11.3)	526 (197)	0.6 (0.3)	5.5 (1.6)	9.1 (3.2)	15.9 (11.0)
	24.5–51	298–759	0.2–1.0	3.9–7.1	5.8–12.2	17.1–23.8
	n = 4	n = 4	n = 4	n = 3	n = 3	n = 3
**Female adults**	41.9 (4.2)	633 (122)	0.4 (0.1)	4.6 (0.58)	8.3 (2.2)	19.3 (3.8)
	37–48.5	420–776	0.2–0.5	3.9–5.6	4.8–11.1	12.6–23.0
	n = 7	n = 7	n = 7	n = 6	n = 6	n = 6

Total carcass weights (TCW) are given in Table 1. The carcasses appeared slightly dehydrated, with eyes sunken. Mild to moderate bloodstained subcutaneous oedema, unrelated to infestation with warble fly larvae, was noticed in 5 cases (18%), affecting ventral and/or lateral parts of neck and head (n = 3), or area around nostrils and upper lip (n = 2).

In most cases the pleural and peritoneal cavities contained small to moderate amounts of bloodstained serous fluid, considered a result of freezing and thawing. In 5 cases (18%) the amount of bloodstained serous fluid was judged as elevated, ranging from 1 to 3 dl in each cavity. Three of these cases also had subcutaneous oedema.

Muscle mass appeared markedly reduced. The results of the muscle index calculations are given in Table 1. The muscle index of calves was significantly lower than for older animals, but there were no significant differences between male and female calves and between two-year-old animals and adults.

Three of the four two-year-old females had juvenile uteruses, while the fourth had an enlarged uterus with developed caruncles and dark bloodstained mucous content, consistent with a recent abortion. Three of the seven adult females had a normally developed foetus in uterus, while the others were non-juvenile and non-pregnant.

### Pathological findings of the digestive tract

Molars and premolars were excessively worn in a single two-year old female; otherwise the wearing of the teeth were considered moderate and without clinical significance.

Lesions in the oral mucosa were found in 6 cases (21%), of which 5 had only 1 to 3 focal lesions of 1–10 mm in diameter, which upon microscopical examination were classified as erosions and shallow ulcers. One animal had extensive lesions throughout the oral cavity, and microscopic examination showed unspecific superficial ulcers with local inflammatory reaction, but gave no clue to the cause of the lesions. No erosive or ulcerative lesions were found in pharynx, oesophagus or forestomachs.

Lesions in the abomasal mucosa occurred in 19 completely emaciated carcasses (68%). The lesions were dark red to brownish and most often elongated, 1–3 mm wide and 5–20 mm long. They tended to occur most frequently at the margins of the *plicae spirales *in the corpus region. The severity of mucosal affection was light in 14 cases (less than 10 lesions), moderate in 4 cases (10–20 lesions) and severe in one case (more than 20 lesions). Microscopic examination showed acute coagulative necrosis with little inflammatory reaction, located in the lamina propria only. The occurrence of oral and abomasal lesions showed no interrelationship and no association with age, gender, feeding history or composition of rumen or abomasal content. Two lean control animals showed similar abomasal lesions.

Abomasum contained small amounts (about a spoonful) of sand and gravel in 10 cases, most prevalent in animals on natural pastures.

Slight to moderate hyperaemia in the abomasal mucosa was observed in 2 carcasses (7%), both had normal dry faecal pellets.

### Faecal consistency and faecal egg count

The consistency of the faeces was described as normal dry winter pellets in 20 carcasses, soft pellets in 3 cases, soft clumps in 3 cases and varying degree of diarrhoea in 2 cases.

Parasitological examination of faecal samples from the totally emaciated carcasses and four controls (including three lean, but not completely emaciated animals, and one animal in good condition) showed no significant differences between the groups. *Moniezia *eggs was found in one emaciated yearling (20 epg), coccidia oocysts in 4 of 17 emaciated yearlings and in 1 of 4 emaciated 2-year-old animals (96 ± 50 oocysts per gram), whereas nematode eggs were recovered both in emaciated carcasses (in 14 of 17 yearlings, and 7 of 11 older animals) and in all 4 controls. Epg faeces were low (between 20 and 220) in most infected animals, whereas 3 emaciated carcasses had 360, 400 and 720 epg faeces, respectively. *Nematodirus *sp., *Capillaria *sp., and trichostrongyles eggs were recovered from all herds, whereas *Skrjabinema *sp. eggs were only recovered from Herd B. *Elaphostrongylus rangiferi *larvae (20–100 per gram faeces) were found in faecal samples of 3 emaciated carcasses and in 2 of 4 controls; representing all three herds.

**Figure 1:**
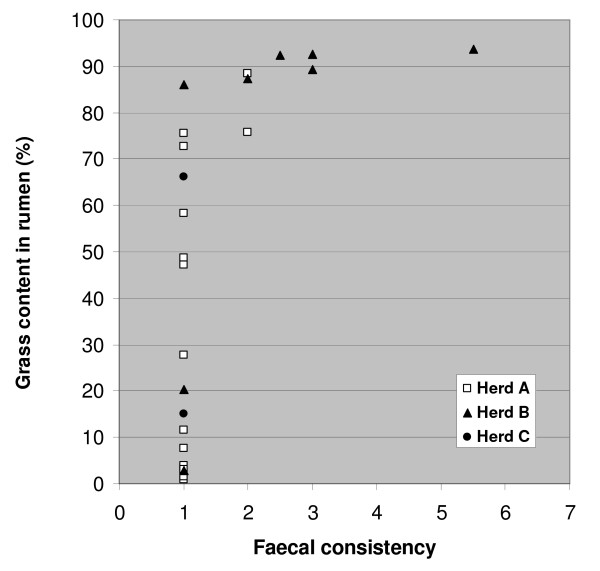
Faecal concistency as a function of grass content in rumen in 25 completely emaciated reindeer carcasses. Faecal concistency: 1 = dry pellets, 2 = soft pellets, 3 = soft lumps, 4–7 = pasty to watery diarrhoea.

**Figure 2:**
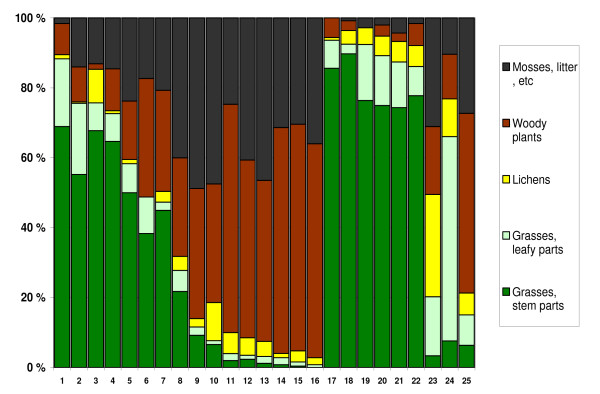
Botanical composition of rumen content in 25 completely emaciated reindeer carcasses from 3 different herds. Herd A: No. 1–16. Herd B: No. 17–23. Herd C: No. 24–25. *Feeding history*: Taken home and fed grass silage for 1 week: No. 24; for 2–4 weeks: No. 1–2 and 17–22. Offered baled grass silage in the field: No. 3–16. Fed for about one day: No 25. No feeding: No. 23.

There was no association between faecal egg count and loose stools, but there was a clear association between silage feeding and faecal consistency. This is expressed in Figure 1, where grass content in rumen (see Figure 2) is used as a measure for silage feeding. Especially the feeding in Herd B was associated with loose stools. Both cases of diarrhoea occurred in this herd, and only one of the seven fed carcasses in Herd B had normal dry pellets in rectum.

### Pathological findings in other organs

Liver weight (Table 1) ranged from 250 to 533 g in yearlings and 298 to 776 g in older animals. The liver was dark, almost black in colour in all cases, and appeared swollen in two cases, one of which was ascribed to extensive acute lesions caused by wandering larvae of *Taenia hydatigena*. This calf also had the peak value of liver weights in yearlings (533 g).

**Figure 3:**
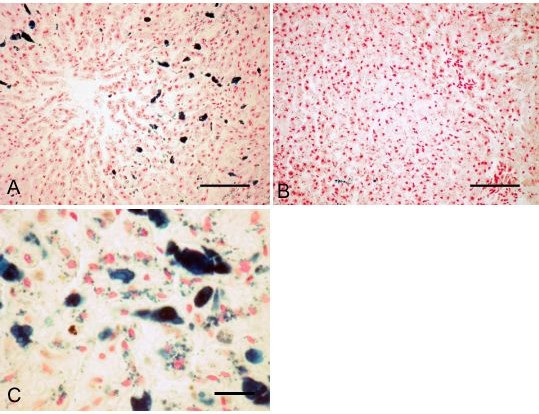
Liver hemosiderosis. A: Completely emaciated adult non-pregnant female. Dark blue lumps of hemosiderin are abundant in sinusoidal macrophages. B: For comparison: Adult pregnant female in good body condition, with scarce amounts of liver hemosiderin. C: Completely emaciated yearling female, showing both lumps of hemosiderin in sinusoidal macrophages and granular deposits of hemosiderin in hepatocytes. Prussian blue. A and B: Bar = 100 μm. C: Bar = 20 μm.

Microscopic examination of liver tissue revealed small, atrophic hepatocytes, and excessive deposits of hemosiderin. The hemosiderin deposits were present as compact lumps in sinusoidal macrophages (Kupffer's cells) (Fig. 3a, 3c), and as granular deposits in parenchymal cells (Fig. 3c). The amount of parenchymal deposits varied from hardly demonstrable to abundant, without any obvious relation to age, gender or reproductive status. The distribution of the hemosiderin deposits showed a distinct perilobular pattern in parenchymal cells, and a less distinct, but still recognisable perilobular pattern in sinusoidal macrophages (Fig. 3a). Microscopic examination of the liver in three lean control carcasses showed that these also had similar or slightly less pronounced hemosiderin deposits, whereas an adult pregnant female in good condition (80 g kidney fat), had only a few sinusoidal macrophages with hemosiderin (Fig. 3b).

Prussian blue staining of liver sections revealed that in addition to the blue-staining hemosiderin granula, there was a variable amount of other brownish granula that did not stain positive with Prussian blue. These granula resembled lipofuscin, and appeared to be of two different types.

**Figure 4:**
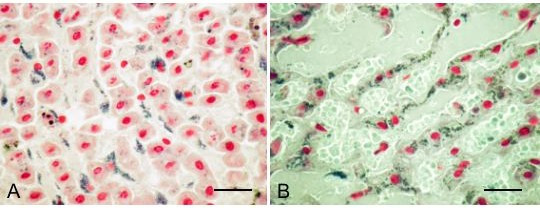
Liver lipofuscinosis. A: Completely emaciated yearling female. Bluish green Schmorl positive granula are present in liver endothelial cells lining the liver trabeculae, but are scarce in hepatocytes. B: Completely emaciated non-pregnant adult female showing abundant Schmorl positive granula in the hepatocytes. Schmorl. Bar = 20 μm.

One type stained positive with Schmorl, but negative with Sudan black, PAS and Long Ziehl-Neelsen. These granula were mainly located in liver sinusoidal endothelial cells (Figure 4a), though granula could be seen also in hemosiderin-laden macrophages. The granula had an even distribution in the liver lobules, and were present in all completely emaciated carcasses, and also in three lean, but not completely emaciated controls. The other type of granula stained positive both with Schmorl and Sudan black, but negative with PAS and Long Ziehl-Neelsen, and were located in hepatocytes (Fig 4b). These granules were absent or only sparsely present in yearlings (Fig. 4a), and occurred in varying amounts in animals two years and older, with some of the adults being the most heavily affected. A lean control carcass was also heavily affected. Even in reindeer with heavy deposits of these granula in liver, no corresponding granula were discovered in heart, brain or adrenals.

The spleen of the completely emaciated carcasses appeared moderately blood-filled and dark, almost black, on the cut surface, and had excessive deposits of hemosiderin in red pulp. Similar or slightly less pronounced hemosiderin deposits were present also in the three lean control animals, but were absent in the adult female in good body condition.

The kidneys were without grossly visible pathological changes. Microscopic examination revealed two cases of mild to moderate multifocal non-purulent chronic interstitial nephritis, which we considered incidental findings without clinical significance. The autolytic changes were more pronounced in the kidneys than in other organs, and three cases were considered too autolytic for histological evaluation. No signs of hemosiderin deposits were seen in renal tubular epithelium in HE-stained sections, and Prussian blue staining of selected sections confirmed that hemosiderin was absent in this tissue.

The urinary bladder was examined in 26 completely emaciated carcasses. The mucosa appeared normal. The bladder was empty in 19 cases, contained small amounts of urine in 4 cases and contained 0.5–1 dl urine in 3 cases. Males had more frequent urine present in bladder (6/10) than females (1/16), possibly explained by post mortem emptying of the bladder, which probably happens more easily in females than males. The urine, if present, was yellow to yellow brown in colour, and contained small greyish flakes in two cases.

The lungs were generally soft and moderately congested with blood. Foam was present in lower trachea and main bronchi in 10 cases (36%). Four carcasses were noted to have firm, non-collapsing lungs. These were later diagnosed to have subacute to chronic diffuse granulomatous pneumonia due to lung nematodes. Rumen content was found in trachea and main bronchi in one case that was euthanized in moribund state. Small amounts of rumen content were seen in upper trachea of two additional cases. Prussian blue staining of selected sections showed that some alveolar macrophages contained hemosiderin. In addition weak diffuse positive staining was seen in alveolar endothelial or epithelial cells.

### Warble fly and throat bot larvae

Normally developed warble fly larvae were found in the skin of 16 completely emaciated carcasses (57%), including 6 of 17 yearlings, 3 of 4 two-year-old animals, and all adult animals. In addition, small, rudimentary 6–7 mm long warble fly larvae was found in the skin of 6 yearlings and one two-year-old animal. Lack of warble fly larvae and occurrence of rudimentary larvae could be attributed to antiparasitic treatment with ivermectin (information from the reindeer herder). The average amount of larvae found in carcasses with normally developed warble fly larvae was 124 (SD = 65, n = 16), with a slightly higher mean in yearlings (127, SD = 78,5, n = 6) than in adults (112, SD = 54, n = 7). Peak value was 298 larvae in a yearling. Warble fly larvae were also found in the two adult control carcasses (63 and 182 larvae respectively).

Throat bot larvae were found in 9 emaciated cases (32%). The average number of larvae was 58 (SD = 55) with heavy infestation (>100 larvae) seen in three animals, one adult female and two yearlings. One adult control contained 37 throat bot larvae. All animals with throat bot larvae also had warble fly larvae infestation.

### Bacteriology

Bacteriological examination of lung, liver and kidney tissue of emaciated carcasses were either negative (lung: 56%; liver: 59%; kidney: 63%) or showed upgrowth of an unspecific flora containing *Clostridium *sp. and/or *Escherichia coli*. *Mannheimia haemolytica *was isolated from lung of one carcass. Histological examination of lung tissue from this animal showed moderate infestation of nematode larvae and eggs, but no tissue inflammation.

### Vitamin E and selenium in liver tissue

Mean liver vitamin E (α-tocopherol) content was 5.41 μg/g wet weight in yearlings (SD 1.77, range 2.8–8.6, n = 19), and 9.71 μg/g in older animals (SD 5.40, range 2.9–18.5, n = 12). Mean liver selenium content was 0.31 μg/g wet weight in yearlings (SD 0.10, range 0.18–0.61, n = 19), and 0.38 μg/g in older animals (SD 0.15, range 0.21–0.73, n = 12).

### Gastrointestinal fill

Ruminoreticular weight (wet weight of rumen and reticulum with content; Table 1) showed large variation between animals, ranging from 1.9 to 7.3 kg in yearlings and 4.8 to 12.2 kg in older animals. The difference between yearlings and older animals was statistically significant (p < 0.05). When ruminoreticular weight was expressed as percentage of TCW there were no significant differences between age groups, but still a large variation, ranging from 12.3 to 26.9% of TCW.

### Analysis of rumen content

Results of botanical analysis of rumen content of completely emaciated carcasses are given in Figure 2. The most significant result of this analysis was how clearly rumen content did reflect silage feeding. In herd B, where 6 out of 7 analysed animals had been fed grass silage for 2–4 weeks, the rumen content was highly dominated by grasses (mean 90%, range 86–94%). In herd A, where the animals had been offered silage balls in the field, some animals had high content of grasses in rumen, while others had very little, indicating that they had not eaten the offered silage.

Another significant finding in the botanical analysis was the leaf:stem ratio in the grass fraction. The 13 animals in herd A and B with more than 40% grasses in rumen (i.e. they had probably eaten silage), had a mean of 29% leaves/71% stems in the grass fraction, while 7 animals in the same herds with less than 10% grass in rumen had a mean of 58% leaves/42% stems. The 2 carcasses in herd C showed no such difference. In this herd leaves dominated the grass fraction, despite up to 66% grass in rumen.

Animals with low content of grasses in rumen showed higher content of woody plants, mosses and litter (Figure 2). The amount of lichens in rumen showed a median value of 4.2%, and 25 out of 26 animals had less than 11% lichens in rumen. Lichen content showed no systematic association with other botanic components.

Results of chemical analysis of rumen content are shown in Table 2. The chemical composition showed no major influence by gender or age, but was clearly influenced by the botanical composition of the rumen content. For this reason the carcasses in Table 2 are separated into two groups according to their rumen grass content determined by the botanical analysis. Differences in the chemical composition between high-grass and low-grass content were statistically significant for most parameters (p < 0.05), except cellulose (p = 0.06) and crude protein (p = 0.91).

**Table 2:** Chemical analysis of rumen content of 19 completely emaciated reindeer carcasses. The carcasses are grouped according to their content of grass in rumen, which in turn is related to whether the animals were fed grass silage or not. Chemical composition is given as % of dry matter (mean and SD). Asterisk (*) denotes statistically significant differences (p < 0.05).

**Grass content in rumen**	**Dry matter (%)**	**Ash**	**Crude protein**	**Hemicelluloses (NDF-ADF)**	**Celluloses (ADF-ADL)**	**Lignin (ADL)**
**Mean**: 76% **Range**: 47–94% (n = 11)	14.1 (3.3)	11.5 (1.8)	22.7 (3.8)	22.9 (3.0)	16.3 (2.4)	15.9 (5.2)
**Mean**: 9% **Range**: 1–28% (n = 8)	19.1* (3.4)	8.9* (1.6)	22.5 (2.8)	19.4* (2.6)	14.4 (1.0)	22.9* (3.1)

There were no strong correlations between ruminoreticular weight and chemical or botanical parameters in the rumen content.

## Discussion

Emaciation may be caused by lack of feed or by different forms of chronic disease. In the present study, chronic disease was observed in some cases, in the form of heavy parasitic infestation with warble fly larvae, throat bot larvae and nematodes in the lung (*Dictyocaulus *sp. and/or *Elaphostrongylus rangiferi *larvae). However, heavy parasite burden was not a consistent finding in completely emaciated carcasses, and occurred also in control animals. So even though the parasite burden have contributed to emaciation in some cases, we still consider the parasites only as modifying factors, and regard the main cause of emaciation to be lack of appropriate amounts and/or appropriate quality of feed. Analysis of vitamin E and selenium in liver showed values within the normal range of sheep and cattle [12], thus eliminating these factors as a contributing cause of chronic wasting.

Some of the main pathological findings in the completely emaciated carcasses may be seen simply as an aggravation of normal physiological changes associated with sub-maintenance feed intake during winter. These include weight loss and muscle atrophy, which commonly occur during wintertime, but is drawn to the extremes in the completely emaciated carcasses. The carcass weights in the present material comprised only 50–70% of normal carcass weights reported [13-17], and the muscle indexes of calves comprised 50–60% of the lowest values recorded by Aagnes and Mathiesen [16].

Liver hemosiderosis and lipofuscinosis may also be regarded as an aggravation of otherwise normal physiological processes associated with a catabolic state. Diffuse hemosiderosis is a common finding in emaciated animals [1], and increased amount of liver iron in winter has been reported in Svalbard reindeer [18,19]. These reports conclude that the liver siderosis is a result of catabolism of blood and lean tissue during a period of sub-maintenance food intake.

Lipofuscin pigments are a more heterogenous group of pigments resulting from peroxidation of subcellular membranes. Its occurrence is commonly associated with age and/or severe malnutrition [20]. Ågren and Rehbinder [3] report abundant lipofuscin granules in hepatocytes of emaciated reindeer. In our material, we identified two lipofuscin-like pigments that differed in staining properties (Sudan black negative versus positive), cell localisation (liver endothelial cells versus hepatocytes) and age distribution (all ages versus elder animals). Neither of the pigments occurred exclusively in completely emaciated animals, indicating that the pigments perhaps should be considered more as a general sign of catabolism rather than a sign of severe undernourishment. The pigments in hepatocytes showed a trend towards age accumulation, consistent with the common apprehension of lipofuscins as being non-degradable residues of membrane breakdown.

In contrast to weight loss, muscle atrophy, hemosiderosis and lipofuscinosis, the finding of subcutaneous oedema and/or increased amount of serous fluid in body cavities is not an extension of normal physiology, and we assume the oedema and body cavity effusions to be caused by hypoproteinaemia. Low blood protein is reported in reindeer in winter, due to protein deficiency in the diet [21]. In our study, we measured very low levels of crude protein (22–23% of dry matter) in the rumen content of the emaciated carcasses. Generalised oedema due to low protein intake is seen in extreme in the human disease called "kwashiorkor" [20]. Even though winter inanition of reindeer must be considered balanced (lack of both energy and protein), it is not surprising that oedema/effusions due to hypoproteinaemia occur in late stages in some animals.

Abomasal lesions are another finding that is not related to normal responses to lack of feed. In the present material, abomasal lesions were superficial, and appeared acute. This type of lesions is a relatively common finding in reindeer, and its relation to stress is well documented [22-24]. This is consistent with our findings, as there was no association between abomasal lesions and other factors that we controlled. The frequency and appearance of the lesions indicate that they may arise immediately (within 24 hours) before death, indicating that a response equivalent to the stress response is elicited in the reindeer prior to death.

In our material we observed lesions in the oral mucosa in 6 cases (21%). However, 5 of these cases had 1–3 small focal lesions that most likely could be caused by sharp plant spicules or alike, which are a common cause of oral lesions in cattle and sheep [12]. One single calf had a more widespread ulcerative stomatitis. Body fluid taken from the carcass was negative for antibodies against herpesvirus [25]. Thus, the stomatitis may be related to the generally poor condition of the animal, but may also be related to the fact that this particular calf had a swollen liver with severe infestation of *Taenia hydatigena *larvae.

The absence of diarrhoea in our material is the single finding that differs most clearly between the present material and previous reports. We found diarrhoea in only 2 cases (7%) in our material, while Westerling [26] reports diarrhoea as a main clinical finding before starvation death in reindeer during emergency feeding in winter. Furthermore he reports diarrhoea in all of 12 reindeer yearlings that died during winter-feeding with the subsequent diagnosis "complete emaciation" [2]. We believe that the clue to the understanding of this apparent discrepancy lies in the feeding. Westerling autopsied six of the twelve reindeer yearlings that died with diarrhoea, and reported accumulation of coarse undigested fodder in the digestive tract [2]. In our material we observed a relationship between loose stools/diarrhoea and grass content in rumen (Figure 1), and we suggest that ruminal indigestion caused by feeding of roughage, without sufficient adaptation, is the cause of diarrhoea in starved reindeer that subsequently succumb with the diagnosis of complete emaciation. The nature of the feeding (supplementary or full ration) and the quality of the feed would be expected to influence the ruminal digestion. In our material only the feeding in Herd B led to some cases of loose stools/diarrhoea, and based on our data we may conclude that inanition and emaciation in itself does not lead to diarrhoea in reindeer.

Ågren and Rehbinder [3] also reported diarrhoea in all 5 cases of reindeer that succumbed with the subsequent diagnosis of complete emaciation. When compared to our material their pathological findings differ in many aspects from our findings in winter inanition. Besides from the diarrhoea, the authors reported pronounced oedema and congestion in the abomasal mucosa, whereas in our material mild hyperaemia and oedema of the abomasal and intestinal mucosa occured only sporadically (2 cases of 28). Furthermore Ågren and Rehbinder described mucosal erosions in the mouth and oesophagus of all cases, while comparable lesions were only observed in the oral mucosa of a single yearling in the present study. They also reported disturbances of pelt shift in all cases, and osteoporosis in one case, both changes that were not recognized in our material. These differences may point towards the possibility that the emaciation reported in Ågren and Rehbinder [3] is not primarily due to deficit of energy or protein, like we see in winter inanition, but may be secondary to other causes, as is also discussed by the authors.

### Why did the supplementary feeding not prevent fatal inanition?

Many of the animals in our study had been offered feed the last weeks before they died, and an important question is why the animals died, despite feeding. The present data does not give a definite answer to this question, as the study was planned as a preliminary study on mortality in reindeer, and not designed to examine the digestive system of reindeer during inanition. It was not suspected in advance that the vast majority of carcasses would end up with inanition as the main diagnosis, and it was even less expected that many of these animals had been subjected to more or less intensive feeding the last weeks before they died. Thus, crucial information about the feed used (amount and quality) are lacking. However, we find reason to believe that the quality of the feed is the most important factor in explaining the "starvation despite feeding paradox." Reindeer has a limited ability to digest fibrous feed, and research on reindeer digestive physiology has documented the great importance of the quality of hay and silage when used as feed for reindeer [14-16,27-30]. The botanical analysis of rumen content in our material showed that silage-fed animals had a high proportion of grass stem in their rumen content. This may indicate that the grass used to produce the silage have been harvested at a comparatively late stage of development, resulting in silage with a high fraction of stems with low digestibility. A reduced ruminal microflora in the emaciated and often partly starved reindeer may probably contribute to a failing ruminal digestion.

## Competing interests

The author(s) declare that they have no competing interests.

## Authors' contributions

All authors participated in the planning of the project. KAR was the leader of the project from which the study arose. TDJ, KKS and TM planned and performed the autopsies, histological and bacteriological examinations and feacal egg counts, and collected all samples for further analyses. Statistical analyses were done by TDJ. SDM initiated the chemical and botanical analysis of rumen content, and KAR initiated the analysis of liver vitamin E and selenium. TDJ were the main author, with contributions from the other authors. All authors read and approved the final manuscript.
